# Inhibitory Activity of 4-*O*-Benzoyl-3′-*O*-(OMethylsinapoyl) Sucrose from *Polygala tenuifolia* on *Escherichia coli* *β*-Glucuronidase

**DOI:** 10.4014/jmb.2108.08004

**Published:** 2021-09-10

**Authors:** Jang Hoon Kim, Le Ba Vinh, Mok Hur, Sung-Cheol Koo, Woo Tae Park, Youn-Ho Moon, Yoon Jeong Lee, Young Ho Kim, Yun-Chan Huh, Seo Young Yang

**Affiliations:** 1 Department of Herbal Crop Research, National Institute of Horticultural and Herbal Science, RDA, Eumseong 27709, Republic of Korea; 2 Institute of Marine Biochemistry(IMBC), Vietnam Academy of Science and Technology(VAST), Hanoi 100000, Vietnam; 3 College of Pharmacy, Chungnam National University, Daejeon 34134, Republic of Korea; 4 Department of Pharmaceutical Engineering, Sangji University, Wonju 26339, Republic of Korea

**Keywords:** *β*-glucuronidase, *Polygala tenuifolia*, uncompetitive inhibitor, molecular simulation

## Abstract

Bacterial *β*-glucuronidase in the intestine is involved in the conversion of 7-ethyl-10- hydroxycamptochecin glucuronide (derived from irinotecan) to 7-ethyl-10-hydroxycamptothecin, which causes intestinal bleeding and diarrhea (side effects of anti-cancer drugs). Twelve compounds (1–12) from *Polygala tenuifolia* were evaluated in terms of *β*-glucuronidase inhibition in vitro. 4-*O*-Benzoyl-3′-*O*-(*O*-methylsinapoyl) sucrose (C**3**) was highly inhibitory at low concentrations. C**3** (an uncompetitive inhibitor) exhibited a *k_i_* value of 13.4 μM; inhibitory activity increased as the substrate concentration rose. Molecular simulation revealed that C**3** bound principally to the Gln158–Tyr160 enzyme loop. Thus, C**3** will serve as a lead compound for development of new *β*-glucuronidase inhibitors.

## Introduction

Gut microbes obtain the carbon sources necessary for survival by hydrolyzing plant polysaccharides. Among products, it is also included glucuronic acid decomposed by the glucuronides process [1]. β-Glucuronidase (E.C. 3.2.1.31) is a member of the lysosomal glycosidase family found in mammalian tissues, plants, insects, and microbes [2]. The enzyme hydrolyzes the glucuronic acid links of glycosides to yield aglycone and free glucuronic acid [3]. Bacterial *β*-glucuronidases are 45% homologous to the human enzyme and catalytic residues in active site composed of Glu413 (catalytic acid) and Glu504 (catalytic nucleophile) [4]. The *Escherichia coli* *β*-glucuronidase has been crystallized by Jain in 1996; two monomers each of 597 amino acid residues form an asymmetric dimer [3,4]. This enzyme is necessary for the digestive process in our small intestine, but when taking anticancer drug, it sometimes converts that to have undesirable effects. [1, 5]. Irinotecan (an anticancer drug [5]) is metabolized by human carboxylesterases and UDP-glucuronosyltransferase to 7-ethyl-10-hydroxycamptothecin glucuronide [5, 6], which is in turn hydrolyzed by bacterial *β*-glucuronidase in the small intestine to 7-ethyl-10-hydroxycamptothecin. This is toxic to intestinal epithelial cells, and triggers diarrhea [4, 5]. Thus, *β*-glucuronidase has been targeted to relieve the irinotecan-induced side effects [3,7]. In recent studies, scutellarein (competitive), luteolin (competitive) amoxapine (uncompetitive), sanggenon C (mixed), and kuwanon G (mixed) have been identified as this enzyme inhibitors [5, 8].

*Polygala tenuifolia* (*P. tenuifolia*) is an herb of the Polygalaceae family; the roots are termed “Yuan-Zhi” in Chinese [9], and have been traditionally used to enhance memory and treat cognitive dysfunctions [10]. The chemical components include saponins [11]; phenylpropanoids [12]; and xanthones [13] such as presenegenin [11], tenuifoliside A [12], and polygalaxanthone III [13]. A heteropolysaccharide in a hot water extract of *P. tenuifolia* reduced the expression of tumor necrosis factor-α and interleukin-6 [10]. Tenuifoliside A exhibited an anti-inflammatory effect, reducing the expression of the p-c-Jun N-terminal kinase of the mitogen-activated protein kinase pathway [14].

## Materals and Methods

### General Experimental Procedures

4-Nitrophnyl-*β*-glucuronide(PNPG, N1627), *β*-glucuronidase(G7396) and D-saccharic acid 1,4-lactone(DSA, S0375) were purchased from Sigma-Aldrich (USA).

### β-Glucuronidase assay

β-Glucuronidase assay was performed as described previously with modified method [15]. Briefly, 130 μl of the enzyme (~ 31 U/ml) in 200 mM phosphate buffer (pH 6.8) was mixed to 96-well plates containing 20 μl of MeOH or compound dissolved in MeOH. 50 μl PNPG (4 mM for inhibition assay, 0.62 to 20 mM for enzyme kinetics) was diluted into the mixture in order. After initiating enzyme reaction at 37°C, the amount of 4-nitrophneol from PNGP were recorded at 405 nm UV-Vis photometer for 20 min. The inhibition ratio was calculated according to the following equation:

Inhibitory activity (%) = [(Δcontrol-Δsample)/Δcontrol]×100 (1)

Where control and sample were the intensity of control and inhibitor after 20 min, respectively.

$${\text{y = y}}_{\text{0}}\text{+[(a×x)/(b+x)]}$$ (2)

where y_0_ is the minimum value on the y-axis, a denotes the difference between maximum and minimum values, and b refers to the x value at 50%.

### Homology Modeling

The targeted sequence of *β*-glucuronoidase (pdb ID: 6LEL) was simulated for homology modeling at Swiss-model (University of Basel, Switzerland). The predicted 3D model was built stably through the process of energy minimization using Gromos96 45a3 force field (Schyler JCC 2001 22 1205) in Gromacs 4.6.5(Stockholm University, Sweden). The corresponding product was evaluated the stability through Ucla-DOE LAB-SAVES v6.0.

### Molecular Docking

Molecular docking was performed by the Autodock 4.2 program (USA). 3 Dimensional inhibitor structure was built by MM2 minimization. Single bond of this was flexibly assigned by using torsion tree of Autodocktools. pdb File of *β*-glucuconidase by homology modelling was added in hydrogens, and then charged with compute gasteiger charges. The grid size was set to include the loop around the active site for molecular docking. The inhibitor (3) was docked into the grid with default values of genetic algorithm parameters excepting for number of GA (runs: 100) and maximun number of evals (25,000,000). The simulation products were presented with Ligplot (UK) and Chimera (USA).

### Molecular dynamics

Molecular dynamics (MD) was carried out using the GROMACS version 4.6.5. The 3D structure for MD simulation was extracted from 2^nd^ autodock score docking pose. .gro and .itp File formats of structure ligand were built to the GlycoBioChem PRODRG2 server. A Gro file of the receptor, produced by pdb2gmx utility with Gromos96 45a3 force field (Schyler JCC 2001 22 1205) of Gromacs, was modified by appending the ligand information. The complexes of inhibitor 3 was eluted with cubic default size and cubic size of 12 × 12 × 12 by the addition of six Cl– ions, respectively. Their energy minimization was stabilized up to 10.0 kJ/mol in steepest descent minimization. The receptor–ligand complex was sequentially subjected to NVT equilibration at 300 K, NPT with Particle Mesh Ewald for long-range electrostatics at 1 bar and MD simulation for 20 ns.

## Results

### β-Glucuronidase inhibition assay

**Fig. 1:**
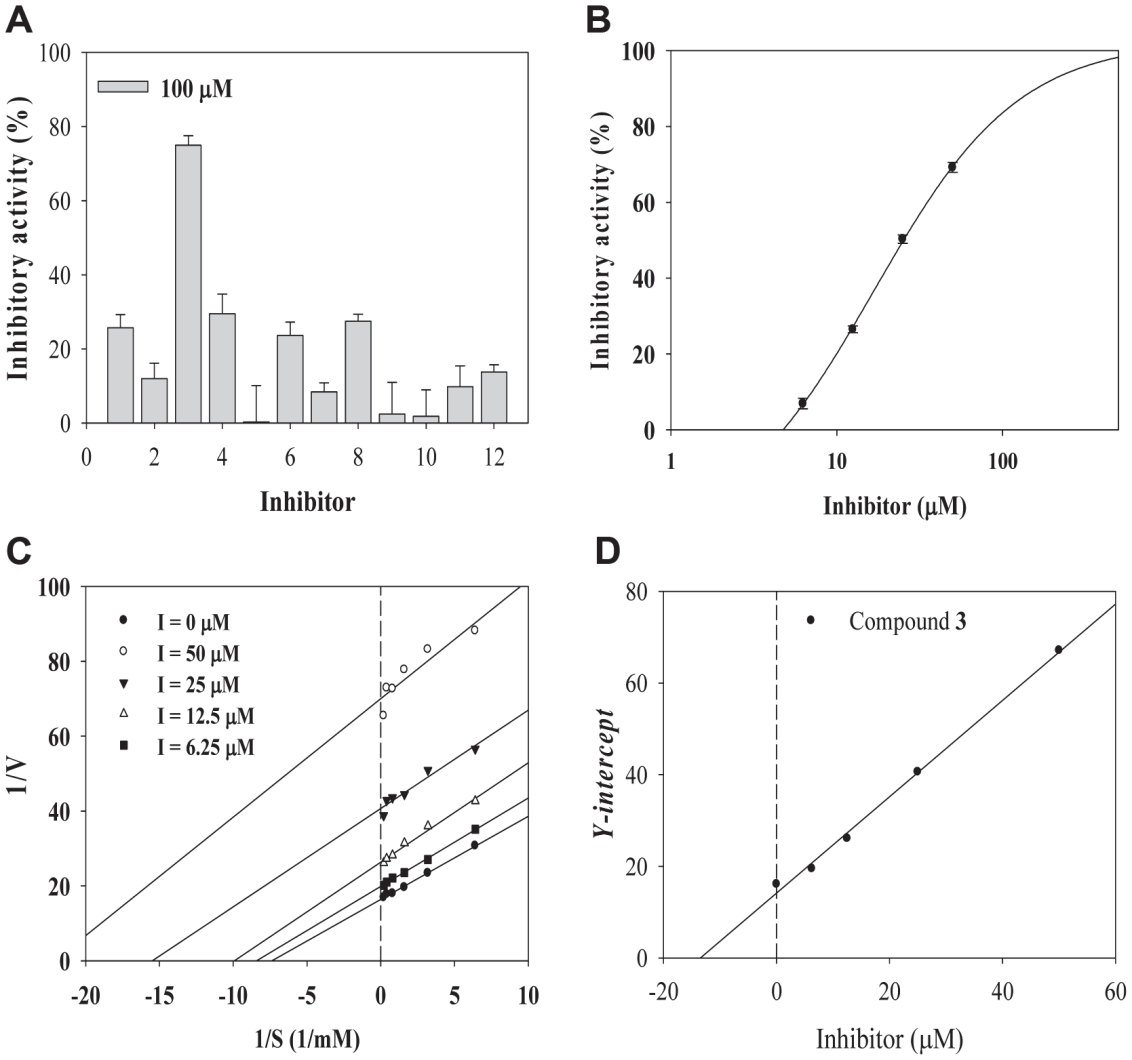
Inhibitory activity of compounds 1-12 (**A**) at 100 μM on *β*-glucuronidase, IC_50_ value of C**3** (**B**), Lineweaver-Burk (**C**) and secondary (**D**) plots of C**3**.

We sought natural *β*-glucuronidase inhibitors. A methanol extract of *P. tenuifolia* exhibited an inhibitory activity of 56.5 ± 4% at a concentration of 100 μg/ml (Eq. 1). Thus, we identified the relevant compound, performed molecular docking, and evaluated the molecular dynamics. Twelve components were tested (**1**: 3′-*O*-(*O*-methylferuloyl)sucrose, **2**: sibiricoseA5, **3**: 4-*O*-benzoyl-3′-*O*-(*O*-methylsinapoyl)sucrose, **4**: tenuifoliside A, **5**: 6-*O*-(*O*-methyl-p-benzoyl)-3′-*O*-(*O*-methylsinapoyl)sucrose, **6**: arillanin A, **7**: 6,3′-di-*O*-sinapoylsucrose, **8**: polygalasaponin XXXII, **9**: desacylsenegasaponin B, **10**: onjisaponin B, **11**: polygalasaponin XXVIII, **12**: platycodin D) [16, 17]. Compound **3** (C**3**) exhibited 74.9 ± 2.6% inhibition at 100 μM and dose-dependent inhibition from 6.2 to 50 μM (Fig. 1A). The IC_50_ value was 24.9 ± 0.8 μM (Eqs. 1 and 2; Table 1). The positive control was D-saccharic acid 1,4-lactone (IC_50_ 19.6 ± 2.4 μM). The initial velocities (*v*_0_ values) of *β*-glucuronidase conversion at inhibitor concentrations of 6.25, 12.5, 25, and 50 μM were measured at substrate concentrations from 0.15 to 5 mM. Fig. 1C shows the Lineweaver–Burk plots. The family of straight lines are of identical slope and cross the x- and y-axes at different points. Thus, inhibition was uncompetitive. The secondary plot shows that the inhibition constant (*k*_i_) was 13.4 μM.

### Homology Modeling

**Fig. 2:**
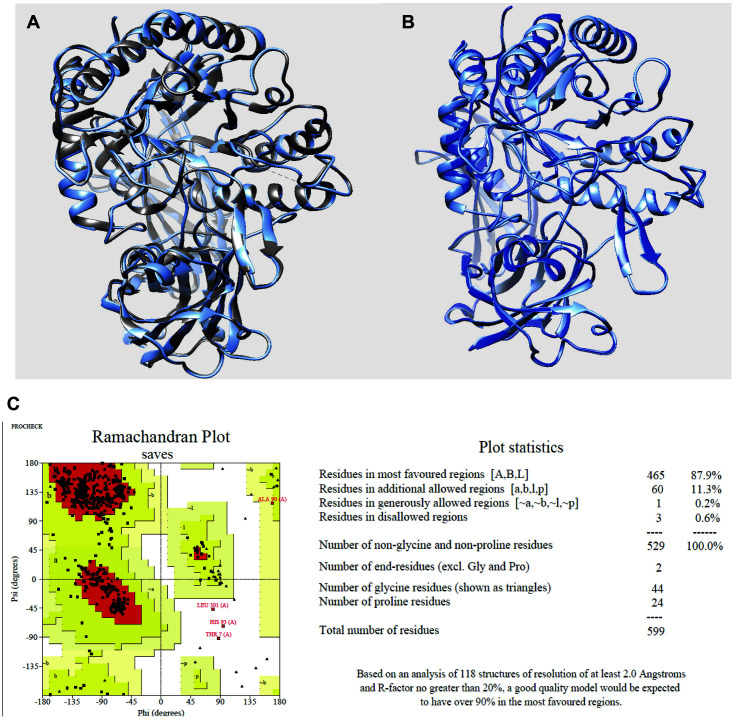
(**A**) Predicitve model (cornflower blue) aligned to the template (gray; pdb ID: 6LEL). (**B**) Overlapped 3D structures of predictive model (cornflower blue) and energy minimized model (blue). (**C**) Ramachandran plot of the main-chain dihedral angles (φ and ψ).

The 3D structure of *β*-glucuronidase (pdb id: 6lel) is missing the amino acids Val206–Ala208 and Glu366–Asn369 (Fig. 2A). Thus, we used homology modeling to build the full 3D structure (Figs. 2A, 2B and S1). The predicted structure was modeled using the -1.52 QMEAN value of the Swiss-Model server (Fig. S1). PROCHECK software was employed to evaluate stereochemical stability (by drawing Ramachandran plots). It was estimated that 99.4% of φ and ψ residue stereochemistries were in the allowed region (most favored regions: 87.9%; additional allowed regions: 11.3%; generously allowed regions: 0.2%; disallowed regions: 0.6%) (Fig. 2C). Finally, as the model had been rebuilt using a template, the GROMOS96 45a3 force field was minimized to stabilize the amino acids (Fig. 2B).

### Molecular Docking and Dynamics

**Fig. 3:**
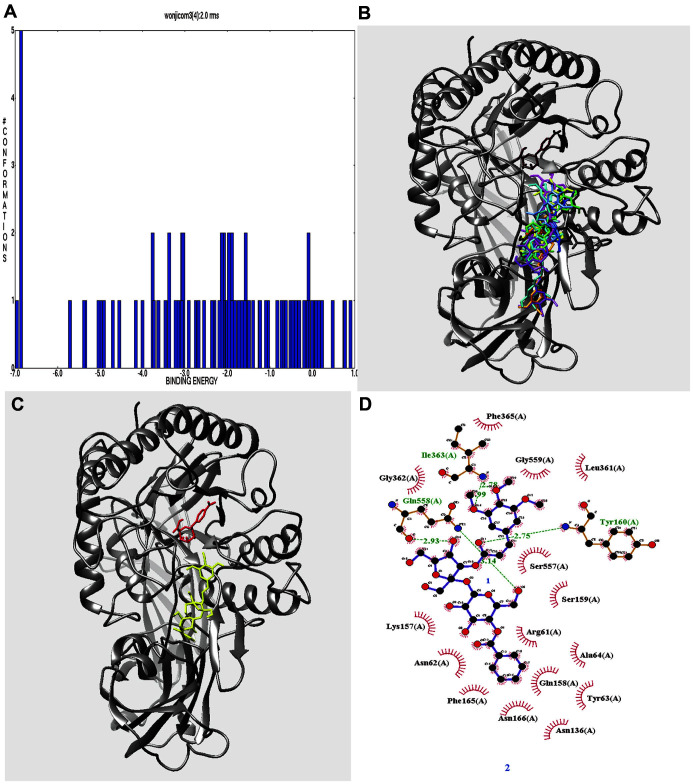
(**A**) Clustering ranks and (**B**) overlapping 3D structure results of the top 10 autodock scores, and (**C**) docking pose (red: substrate, yellow: ligand) and (**D**) hydrogen bonds of 2 rank.

**Fig. 4:**
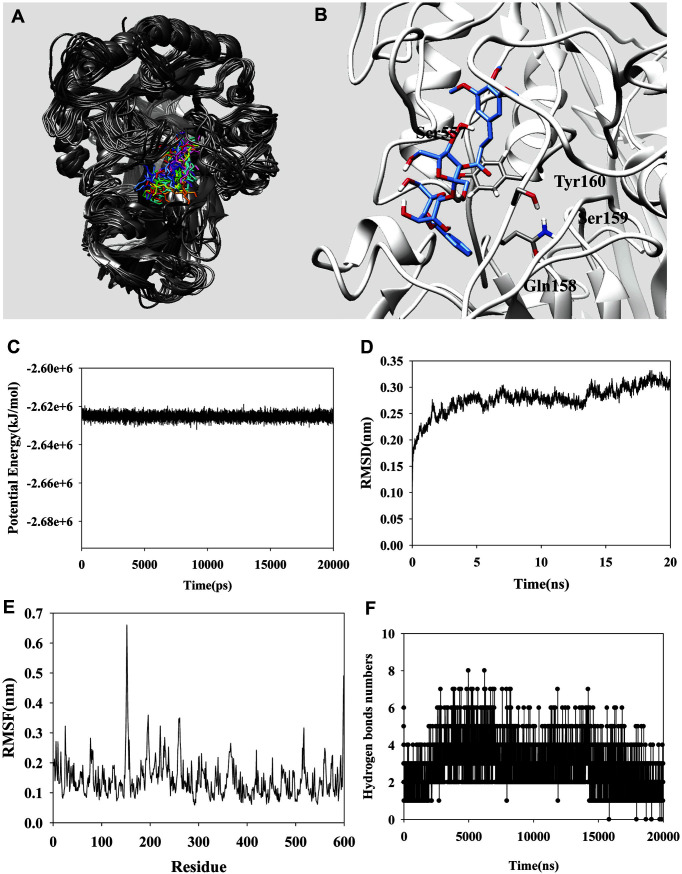
(**A**) The suerpositions of C**3** into *β*-glucuronidase for the simulation time (red: 0, orange: 2, yellow: 4, green: 6, forest green: 8, cyan: 10, light sea green: 12, blue: 14, cornflower blue: 16, purple: 18, hot pink: 20 ns). (**B**) Key amino acids relating hydrogen bonds with inhibitor. The potential energy (**C**), RMSD (**D**), RMSF (E), and hydrogen bond numbers(F) of C**3** with enzyme.

Docking was used to identify the binding site of the (uncompetitive inhibitor) C**3**, which was docked to the enzyme after substrate docking. Inhibitor binding was tighter after the substrate was bound, suggesting that C**3** might bind to a loop adjacent to the active site. A grid including such a loop was evaluated in terms of C**3** binding (25,000,000 times). As shown in Fig. 3A, the top 100 autodock scores were extracted and the clusters analyzed. The lowest autodock score was -6.96 kcal/mol, but there was no cluster (Figs. 3A and 3B). Therefore, the second autodock score (-6.86 kcal/mol) (associated with a cluster) was selected as the best pose (Fig. 3C and Table 2). The complex features five hydrogen bonds (Tyr160: 2.75 Å; Ile363: 2.78 and 2.99 Å; and Gln558: 2.93 and 3.14 Å)(Fig. 3D). Additionally, the top 10 autodock scores confirmed that C**3** maintained hydrogen bonds with Gln158 and Gln558 (Table 2). Molecular dynamics allows dynamic exploration of ligand-receptor interactions [18]. Our simulation ran for 20 ns; Fig. 4A is the 3D result. Four amino residues (Gln158, Ser159, Tyr160, and Ser557), not identified via molecular docking, were involved in hydrogen bonding (Fig. 4B and Table 3). C**3** bound principally to a loop of three amino residues (Gln158, Ser159, and Tyr160). As shown in Fig. 4A, the two loops where C**3** bound moved irregularly, compromising catalytic action. The potential energy of the complex was −2.62 × 10^6^ kcal/mol (Fig. 4C). The root-mean-square deviation (RMSD) was ~ 0.3 nm (based on the protein) (Fig. 4D). The root-mean-square fluctuations (RMSFs) were under ~ 0.35 nm except for Glu152 and Asn153 (~ 0.66 nm) (Fig. 4E). C**3** binding increased the fluidities of the affected loops. C**3** bound via two-to-four hydrogen bonds (Fig. 4F) to Gln158, Ser159, Tyr160, and Ser557 of the flexible complex (Table 3). Thus, molecular dynamics revealed amino residues involved in hydrogen bonding that were not identified by docking.

## Discussion

Recently, the natural xanthones demethylbellidinfolin and gentisin [2], and the natural flavonoids scutellarein and luteolin [4], were shown to be mixed and competitive inhibitors (respectively) of *β*-glucuronidase. Enzyme activity decreased as the substrate concentration increased. On the contrary, C**3** exhibited a low inhibitory activity when the level of the toxic material was low, but a high activity when the level rose. Moreover, the structure of C**3** was similar to that of cryptochlorogenic with good affinities toward *β*-glucuronidase [19]. Most synthetic organic compounds, thus benzimidazole analogs [6], 2-arylquinazolin-4(*3H*)-one derivatives [20], and thiadiazole derivatives [21] were also inhibitory in the micromolar range.

Pyrazol[4,3-c]quinolone derivatives, which showed the inhibitory activity on endogenous *β*-glucuronidase of *Eubacteriumps*. and *Peptostreptococcus anaerobius*., downregulated activity of bacterial enzyme in the intestine of mice [22]. 4-*O*-Benzoyl-3′-*O*-(*O*-methylsinapoyl)-sucrose (C**3**) from *P. tenuifolia* inhibited the enzyme of interest at micromolar concentrations in a dose-dependent manner. C**3** was an uncompetitive inhibitor with a *k*_i_ of 13.4 μM. C**3** interacted with two loops (left loop: Gln158, Ser159, Tyr160; right loop: Ser557) near the catalytic site of the remodeled 3D *β*-glucuronidase structure, principally with the three amino acids of the left loop. *In silico*, C**3** suppressed enzyme action by limiting the loop movements required for catalysis. Our study confirmed that C**3** inhibited the binding of enzyme and substrate. To regulate the metabolism of SN38G derived from irinotecan in the small intestine, there were two methods of inhibiting the catalytic reaction of *β*-glucuronidase and the growth of *E. coli* [23]. Thus, this compound should be evaluated for its effect on the growth of bacterial, as well as for its inhibition of catalytic reaction of *β*-glucuronidase in the intestine of mice [23, 24]. If compound **3** has an inhibitory effect on both or one of the former and the latter. A study on the alleviation of the side effects of anticancer drugs by oral administration together with C**3** and irinotecan is needed. Finally, C**3** is a novel *β*-glucuronidase inhibitor, and will attract the attention of chemists and enzymologists interested in *β*-glucuronidase.

## Supplemental Materials

Supplementary data for this paper are available on-line only at http://jmb.or.kr.

## Figures and Tables

**Table 1:** The inhibitory activity of samples on *β*-glcucuronidase.

	Inhibitory activity^a^ (%, 100 μM)	IC_50_ value (μM)	Binding Mode (ki, μM)
1	25.7±3.5	-	
2	12.0±4.1	-	
3	74.9±2.6	24.9±0.8	13.4
4	29.5±5.3	-	
5	0.2±9.8	-	
6	23.6±3.6	-	
7	8.4±2.4	-	
8	27.4±1.9	-	
9	2.4±8.5	-	
10	1.8±7.1	-	
11	9.8±5.6	-	
12	13.7±2.0	-	
DSA^b^	-	19.6±2.4	
MeOH ex	56.5±4.5 (at 100 μg/mL)		

^a^all sample examined in a set of triplicated experiments.

^b^Positive control

**Table 2:** The top 10 autodock scores of C**3** with 3D structure enzyme.

Ranks	Autodock score (kcal/mol)	Hydrogen bonds (Å)
1	-6.96	Ala64(3.24), Asn62(2.47), Tyr160(3.32), Gln158(3.16), Gln558(2.85)
2	-6.86	Tyr160(2.75), Gln558(2.93, 3.14), Ile363(2.78, 2.99)
3	-6.44	Gln158(2.72), Tyr160(2.67), Ile363(3.07), Lys370(2.89, 3.06)
4	-5.76	Asn62(2.86), Tyr160(2.77), Gln558(2.77)
5	-5.71	Asn62(3.10), Ala64(2.90), Gln158(2.81)
6	-5.69	Lys157(3.05), Gln158(2.51), Gln558(3.04)
7	-5.38	Gln158(3.01), Gln558(2.66,2.95)
8	-5.35	Gln558(2.68)
9	-5.25	Gln158(2.55)
10	-5.04	Asn62(3.35), Lys370(2.64, 2.71), Gln558(3.15)

**Table 3:** Hydrogen bonds analysis of C3 with enzyme at 2 ns intervals form 20 ns.

Time	Hydrogen bonds (Å)
0	Tyr160(2.69), Ile363(3.12,3.25), Gln558(2.85)
2	Lys157(3.12), Ser159(2.45,3.03), Tyr160(2.52)
4	Gln158(2.99), Ser159(2.74), Ser557(2.41,2.57)
6	Ser159(2.56), Tyr160(2.65), Ser557(2.57), Gln558(2.83,3.16), Arg562(2.99)
8	Gln158(3.27), Ser159(2.45), Tyr160(2.60), Ser557(2.64)
10	Gln158(3.08), Ser159(2.37), Tyr160(2.52,3.25)
12	Gln158(3.22), Ser159(2.54), Tyr160(2.53), His162(3.10)
14	Ser159(2.44), Tyr160(2.52,3.13), Ser557(2.54)
16	Tyr160(2.47,3.23,3.34), Ser557(2.81), Gln558(2.80), Arg562(2.94,3.06)
18	Ser159(2.99), Tyr160(2.71)
20	Ser159(3.21), Tyr160(2.55,3.19), Arg562(3.24)

## References

[1] Dashnyam P, Mudududdla R, Hsieh T-J, Lin T-C, Lin H-Y, Chen P-Y (2018). β-Glucuronidases of opportunistic bacteria are the major contributors to xenobiotic-induced toxicity in the gut. Sci. Rep..

[2] Awolade P, Cele N, Kerru N, Gummidi L, Oluwakemi E (2020). Therapeutic significance of *β*-glucuronidase activity and its inhibitors: A review. Eur. J. Med. Chem..

[3] Sun C-P, Yan J-K, Yi J, Zhang XY, Yu Z-L, Huo X-K (2020). The study of inhibitory effect of natural flavonoids toward *β*-glucuronidase and interaction of flavonoids with *β*-glucuronidase. Inter. J. Biol. Macromol..

[4] Wallace BD, Wang H, Lane KT, Scott JE, Orans J, Koo JS (2005). Alleviating cancer drug toxicity by inhibiting a bacterial enzyme. Science.

[5] Weng Z-M, Wang P, Ge G-B, Dai Z-R, Wu D-C, Zou L-W (2017). Structure-activity relationships of flavonoids as natural inhibitors against *E. coli*
*β*-glucuronidase. Food Chem. Toxicol..

[6] Smith NF, Figg WD, Sparreboom A (2006). Pharmacogenetics of irinotecan metabolism and transport: an update. Toxicol. In Vitro.

[7] Cheng KW, Tseng CH, Yang CN, Tzeng CC, Cheng TC, Leu YL (2017). Specific inhibition of bacterial *β*-glucuronidase by pyrazolo[4,3-*c*]quinoline derivatives via a pH-dependent manner to suppress chemotherapy-induced intestinal toxicity. J. Med. Chem..

[8] Wei B, Yang W, Yan Z-X, Zhang Q-W, Yan R (2018). Prenylflavonoids sanggenon C and kuwanon g from mulberry (*Morus alba* L.) as potent broad-spectrum bacterial *β*-glucuronidase inhibitors: Biological evaluation and molecular docking studies. J. Funct. Foods.

[9] Lacaille-Dubois M-A, Delaude C, Mitaine-Offer A-C (2020). A review on the phytopharmacological studies of the genus Polygala. J. Ethnopharm..

[10] Li J, Zhong J, Chen H, Yu Q, Yan C (2020). Structural characterization and anti-neuroinflammatory activity of a heteropolysaccharide isolated from the rhizomes of *Polygala tenuifolia*. Ind. Crops Prod..

[11] Zhang F-S, Zhang X, Wang Q-Y, Pu Y-J, Du C-H, Qin X-M (2020). Cloning, yeast expression, and characterization of a β-amyrin C-28 oxidase (CYP716A249) involved in triterpenoid biosynthesis in *Polygala tenuifolia*. Biol. Pharm. Bull..

[12] Huang C-I, Hu Y, Liu P, Dong X-z, Yu B-y, Mu L-h (2014). Effect of tenuifoliside A isolated from *Polygala tenuifolia* on the ERK and PI3K pathways in C6 glioma cells. Phytomedicine.

[13] Liu J, Liu A, Mao F, Zhao Y, Cao Z, Cen N (2019). Determination of the active ingredients and biopotency in *Polygala tenuifolia* Willd. and the ecological factors that influence them. Ind. Crops Prod..

[14] Kim K-S, Lee D-S, Bae G-S, Park S-J, Kang D-G, Lee H-S (2013). The inhibition of JNK MAPK and NF-κB signaling by tenuifoliside A isolated from *Polygala tenuifolia* in lipopolysaccharide-induced macrophages is associated with its antiinflammatory effect. Eur. J. Pharmacol..

[15] Taha M, Ullah H, Muqarrabun LMRA, Khan MN, Rahim F, Ahmat N (2018). Synthesis of bis-indolylmethanes as new potential inhibitors of *β*-glucuronidase and their molecular docking studies. Eur. J. Med. Chem..

[16] Vinh LB, Kim JH, Lee JS, Nguyet NTM, Yang SY, Ma JY (2018). Soluble epoxide hydrolase inhibitory activity of phenolic glycosides from *Polygala tenuifolia* and *in silico* approach. Med. Chem. Res..

[17] Vinh LB, Heo M, Phong NV, Ali I, Koh YS, Kim YH (2020). Bioactive compounds from *Polygala tenuifolia* and their inhibitory effects on lipopolysaccharide-stimulated pro-inflammatory cytokine production in bone marrow-derived dendritic cells. Plants.

[18] Liew SY, Sivasothy Y, Shaikh NN, Isa DM, Lee VS, Choudhary MI (2020). β-Glucuronidase inhibitors from Malaysian plants. J. Med. Struct..

[19] Chen G-Y, Zhang H, Yang F-Q (2021). A simple and portable method for *β*-glucosidase activity assay and its inhibitor screening based on a personal glucose meter. Anal. Chim. Acta.

[20] Khan KM, Saad SM, Shaikh NN, Hussain S, Fakhri MI, Perveen S (2014). Synthesis and *β*-glucuronidase inhibitory activity of 2-arylquinazolin-4(*3H*)-ones. Bioorg. Med. Chem..

[21] Taha M, Almandil NB, Rashid U, Ali M, Ibrahim M, Gollapalli M (2019). 2,5-Disubstituted thiadiazoles as potent *β*-glucuronidase inhibitors; Synthesis, in vitro and *in silico* studies. Bioorg. Chem..

[22] Cheng K-W, Tseng C-H, Tzeng C-C, Leu Y-L, Cheng T-C, Wang J-Y (2019). Pharmacological inhibition of bacterial *β*-glucuronidase prevents irinotecan-induced diarrhea without impairing its antitumor efficacy in vivo. Pharmacol. Res..

[23] Yang W, Wei B, Yan R (2018). Amoxapine demonstrates incomplete inhibition of *β*-glucuronidase activity from human gut microbiota. SLAS Discov..

[24] Ebuzoeme C, Etim I, Ikimi A, Song J, Du T, Hu M (2021). Glucuronides hydrolysis by intestinal microbial *β*-glucuronidases (GUS) is affected by sampling, enzyme preparation, buffer pH, and species. Pharmaceutics.

