# Supplementary figures and images for: Inhibitory Activity of 4-O-Benzoyl-3′-O-(OMethylsinapoyl) Sucrose from Polygala tenuifolia on Escherichia coli β-Glucuronidase
Source: J Microbiol Biotechnol. 2021 Sep 10;31(11):1576–82. doi: 10.4014/jmb.2108.08004 (PMC9705844; doi:10.4014/jmb.2108.08004)

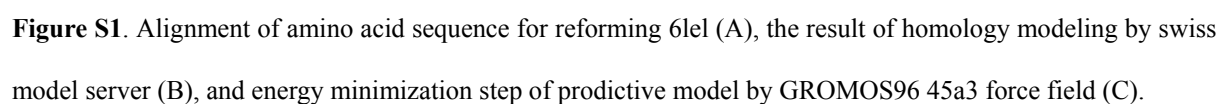

Supplement: Supplementary file 1 [file jmb-31-11-1576-supple.pdf]
